# Conjugated Microporous Polymers Based on Ferrocene Units as Highly Efficient Electrodes for Energy Storage

**DOI:** 10.3390/polym15051095

**Published:** 2023-02-22

**Authors:** Maha Mohamed Samy, Mohamed Gamal Mohamed, Shiao-Wei Kuo

**Affiliations:** 1 Department of Materials and Optoelectronic Science, College of Semiconductor and Advanced Technology Research, Center for Functional Polymers and Supramolecular Materials, National Sun Yat-sen University, Kaohsiung 804, Taiwan; 2 Chemistry Department, Faculty of Science, Assiut University, Assiut 71515, Egypt; 3 Department of Medicinal and Applied Chemistry, Kaohsiung Medical University, Kaohsiung 807, Taiwan

**Keywords:** conjugated microporous polymers (CMPs), triphenylamine, ferrocene, Schiff-base reaction, energy storage

## Abstract

This work describes the facile designing of three conjugated microporous polymers incorporated based on the ferrocene (FC) unit with 1,4-bis(4,6-diamino-s-triazin-2-yl)benzene (PDAT), tris(4-aminophenyl)amine (TPA-NH_2_), and tetrakis(4-aminophenyl)ethane (TPE-NH_2_) to form PDAT-FC, TPA-FC, and TPE-FC CMPs from Schiff base reaction of 1,1′-diacetylferrocene monomer with these three aryl amines, respectively, for efficient supercapacitor electrodes. PDAT-FC and TPA-FC CMPs samples featured higher surface area values of approximately 502 and 701 m^2^ g^−1^, in addition to their possession of both micropores and mesopores. In particular, the TPA-FC CMP electrode achieved more extended discharge time compared with the other two FC CMPs, demonstrating good capacitive performance with a specific capacitance of 129 F g^−1^ and capacitance retention value of 96% next 5000 cycles. This feature of TPA-FC CMP is attributed to the presence of redox-active triphenylamine and ferrocene units in its backbone, in addition to a high surface area and good porosity that facilitates the redox process and provides rapid kinetics.

## 1. Introduction

The pursuit for renewable energy sources, included biomass energy, wind power, solar, geothermal, tidal, marine, and hydraulic, has been elevated to secure the energy requirements in the future. Due to geography, time, and seasons, these renewable sources greatly fluctuate so exploring continuous energy storage systems became necessary. For this reason, fuel cells, conventional capacitors, batteries, and supercapacitors became efficient devices for storing and transferring electrical energy [1,2,3,4,5,6]. Among energy storage/conversion systems, scientists considered supercapacitors (SCs) an alternative energy storage device because they have specific characteristics (i.e., environmental friendliness, speedy charge/discharge time, prolonged lifetime, high power density, and lower input resistance) compared with other individual and hybrid systems [7,8,9,10,11]. According to their advantages, they are employed in some applications related to renewable energy storage, power supply, consumer electronics, voltage stabilization, tools, energy harvesting, microgrid, medical applications, energy recovery, streetlights, and automotive applications [12,13,14]. Based on the principle of SCs energy storage, they are classified into three fundamental categories, electric double-layer capacitors (EDLCs), pseudocapacitors (PCs), and hybrid supercapacitors (HSCs). In EDLCs, charge storage happens among the electrode and electrolyte (electrostatically), while in PCs, it happens by rapid redox interactions (Faradaic reaction) to achieve high capacitance. Finally, HSC stores the energy through both electrostatic and electrochemical reactions [15,16,17,18]. Recently, reports have mentioned the characteristics of the electrode materials that are predicted to determine the efficiency of SCs such as good conductivity, low cost, chemical stability for a long time, environmentally safe, and lower corrosion resistance [19,20,21,22,23,24,25]. The different kinds of electrode materials that are generally utilized for supercapacitor devices are activated carbon, carbon nanotubes, graphite, graphene, metal oxides, porous carbon, metal-doped carbon, and conductive polymers (including, conjugated microporous polymers) [22,26,27].

**Scheme 1:**
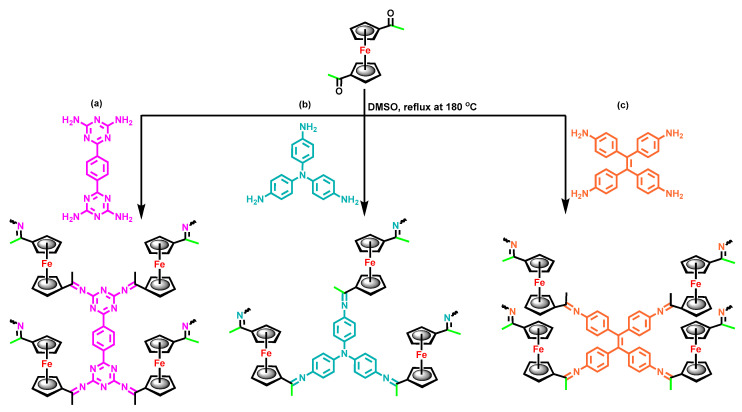
Synthesis of (**a**) PDAT-FC CMP, (**b**) TPA-FC CMP, and (**c**) TPE-FC CMP.

Conjugated microporous polymers (CMPs) are a functional category of the porous organic polymers (POPs) family, offering permanent micropores with π-conjugated electrons extended within their structures. Compared with COFs and MOFs, the amorphous properties of CMPs materials let them utilize more available building blocks in addition to various flexible preparation methods to examine the interaction between the π-conjugate framework and the micropores, so they can be applied in numerous potential fields such as catalysis, energy storage, chemosensors, gas storage-separation, light harvesting, biological uses, and energy conversion [28,29,30,31,32,33,34,35,36,37,38,39,40]. The CMPs family are generally designed via a plethora of coupling reactions (C–C or C–N), for example, Yamamoto, Sonogashira, Suzuki, Schiff base reactions, oxidative polymerizations, Buchwald–Hartwig amination, cyclotrimerizations, in addition to the Chichibabin reaction which was introduced recently to construct different CMPs structures [41,42,43,44,45]. Additionally, the introduction of open cavities such as a ferrocene moiety in the CMPs skeletons will provide them with special properties and be applied in numerous fields [46,47,48,49]. The ferrocene unit is the organometallic component that combines both features of organic and metallic compounds, such as sandwich-style structure and higher electron density, so it acts as an effective electron donor, showing outstanding redox performance. Consequently, when the ferrocene moiety is utilized as a building unit to design polymer structures, it endows new properties to them such as exceptional optical, magnetic, electrical, catalytic, and sensing characteristics, so that Ferrocene-based polymers are exercised in potential applications, such as redox batteries, catalysis, gas sorption, pollutant removal, memory devices, and energy storage, etc. [50,51,52,53,54,55,56,57,58]. Samy et al. prepared BP-FC-CMP with a specific capacitance of 608 F g^−1^ [51]. All the FC CMPs containing redox-active ferrocene moieties that accelerate reversible reactions promoted pseudocapacitance, in addition to the effective motion of the electrolyte over the pores of the CMP materials, participating in enhanced efficiency [59]. In this study, three conjugated microporous polymers (abbreviated as PDAT-FC, TPA-FC, and TPE-FC CMPs) are achieved by incorporating the ferrocene building block with three aryl amines 1,4-bis(4,6-diamino-s-triazin-2-yl)benzene (PDAT), tris(4-aminophenyl)amine (TPA-NH_2_), and tetrakis(4-aminophenyl)ethane (TPE-NH_2_), respectively, through one step of the Schiff base reaction, for testing in supercapacitor application (Scheme 1). The chemical structures of synthesized PDAT, TPA-NH_2,_ and TPE-NH_2_ were confirmed through Fourier transform infrared (FTIR), ^1^H, and ^13^C Nuclear magnetic resonance (NMR) spectroscopy. The chemical structure; thermal decomposition temperature; char yield; Brunauer, Emmett, and Teller (BET) specific surface area; total pore volume; pore diameters; surface morphology; and crystallinity property of the as-prepared PDAT-FC, TPA-FC, and TPE-FC CMPs were carefully investigated and discussed in detail through Fourier transform infrared, solid-state ^13^C NMR spectroscopy, thermal gravimetric analysis (TGA), X-ray powder diffraction (XRD), transmission electron microscopy (TEM), scanning electron microscopy (SEM), and N_2_ adsorption and desorption analyses. Finally, these as-prepared PDAT-FC, TPA-FC, and TPE-FC CMPs were applied as organic electrodes in the three-electrode system, and the electrochemical results revealed that the TPA-FC CMP sample has excellent capacitive performance, with a specific capacitance of 129 F g^−1^ and a capacitance retention value of 96% over the following 5000 cycles. The outstanding characteristics of the TPA-FC CMP are attributed to the existence of redox-active triphenylamine and ferrocene units in its backbone, as well as to the material’s large surface area and excellent porosity, which aid in the redox process and give rapid kinetics.

## 2. Materials and Methods

### 2.1. Materials

Ferrocene, acetyl chloride (CH_3_COCl), aluminum chloride (AlCl_3_), 1,4-dicyanobenzene, 4,4′-diaminobenzophenone, 2-cyanoguanidine, dichloromethane, potassium hydroxide, DMF, Tin powder (Sn), potassium hydroxide (KOH), palladium on carbon (Pd/C) (10 wt%), hydrazine monohydrate, hydrochloric acid (HCl), dimethyl sulfoxide (DMSO), THF, methanol, chloroform, and acetone were gained from different trade resources, including Acros (New Jersey, NJ, USA), Alfa-Aesar (Lancashire, UK) and Sigma-Aldrich (Louis, MO, USA). Tris(4-nitrophenyl)amine (TPA-NO_2_) was prepared according to previous papers [60].

### 2.2. Preparation Method of 1,1′-diacetylferrocene

A mixture of acetyl chloride (CH_3_COCl) (1.39 g, 17.70 mmol) and aluminum chloride (AlCl_3_) (2.50 g, 18.75 mmol) in dichloromethane (CH_2_Cl_2_) (10 mL) was inserted into a stirred suspension of ferrocene (2.00 g, 10.74 mmol) and CH_2_Cl_2_ (30 mL) [51]. Next, all mixture contents were stirred at 40 °C for 8 h. After keeping the system cooled, it was added to cold water (250 mL). The resulting product was filtered, washed well with distilled water, and finally kept in the oven for drying to yield a deep-red product of diacetylferrocene monomer (Scheme S1). FTIR (Figure S1): 3088, 1668, 1391 and 1011 (cyclopentadiene rings). ^1^H NMR (Figure S2a): 4.79, 4.59, 2.28. ^13^C NMR (Figure S2b): 200, 80, 73, 71, 27.

### 2.3. Preparation Method of 1,4-bis(4,6-diamino-s-triazin-2-yl)benzene (PDAT)

A mixture of 2-cyanoguanidine (2.024 g, 24 mmol) and potassium hydroxide (0.562, 10 mmol) inside DMF (80 mL) was added to a suspension of 1,4-dicyanobenzene (0.772 g, 4.6 mmol) in DMF (20 mL). Then, the reaction was homogeneously stirred and refluxed at 150 °C for about 20 hrs under nitrogen. After completing the reaction, the product was washed wholly with ethanol and methanol multiple times, and finally it was dried by putting it in an oven for one day to yield a white powder of PDAT (Scheme S2). FTIR (Figure S3): 3311, 3135, and 1659. ^1^H NMR (Figure S4a): δ (ppm) 8.33, 6.83. ^13^C NMR (Figure S4b): δ (ppm) 170.65, 168.46, 139.96, 127.91.

### 2.4. Preparation Method of Tris(4-aminophenyl)amine (TPA-NH_2_)

TPA-NO_2_ (15.2 g, 0.4 mmol), and 1.0 g of Pd/C (10 wt%) were dissolved in a mixture of 1,4-dioxane and ethanol, and the reaction mixture was heated to reflux under a nitrogen atmosphere in a 250 mL four-neck flask [60]. Then, 80 mL of hydrazine monohydrate was added dropwise into the reaction mixture over five hours. After that, the reaction mixture underwent further refluxing for 20 h. After the complete reaction, the reaction mixture was filtered once it reached room temperature, and the filtrate was then added to 4 L of water. The precipitate was filtered out and dried in vacuo (10.2 g, 89%). The raw material was recrystallized from ethanol to afford gray crystals with a melting point of 246 °C. FTIR (KBr, cm^−1^): 3406, 3336, 1618, 1502, 1331, 1261, 1117, and 829. ^1^H NMR (DMSO-*d_6_*), (ppm): 6.58 (6H, ArH), 6.43 (6H, ArH), 4.72 (6H, NH_2_).

### 2.5. Preparation Method of Tetrakis(4-aminophenyl)ethene [TPE-NH_2_]

Tin powder (Sn) (3.00 g, 25 mmol) was charged slowly into a two-neck flask containing a mixture of 4,4′-diaminobenzophenone (1.00 g, 4.49 mmol) and hydrochloric acid (HCl) (50 mL) [61]. Then, the reaction was left to heat at 70 °C under N_2_. After completing the heating for 7 h, the used solvents were removed by filtration, and the residual solid was washed wholly by using NaOH (1 N), methanol, and hot water. The green powder was put in an oven under a vacuum at 60 °C for drying (Scheme S3). FTIR (Figure S5): 3421, 3357, 3027, 1520. ^1^H NMR (Figure S6a): 6.58, 6.28, 4.84. ^13^C-NMR (Figure S6b): 146, 137, 133, 132, 113.

### 2.6. Preparation of PDAT-FC CMP

**Figure 1:**
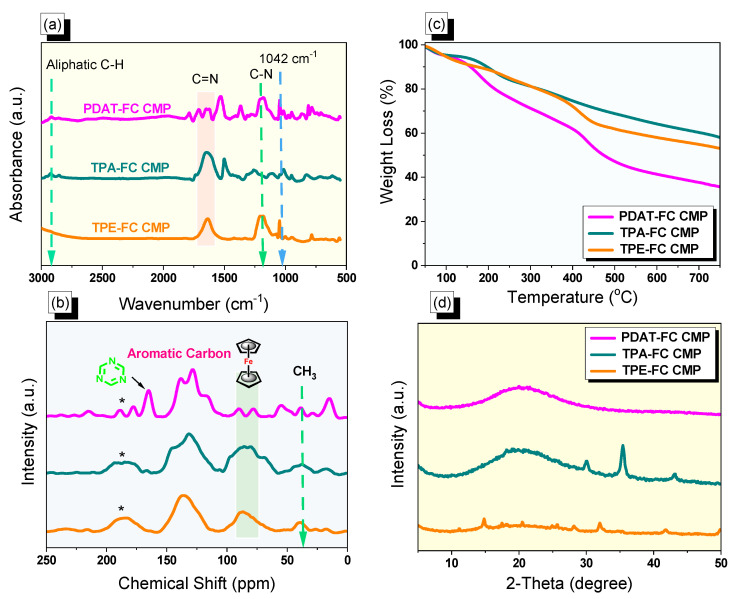
(**a**) FTIR, (**b**) ^13^C NMR solid state, (**c**) TGA, and (**d**) XRD profiles PDAT-FC, TPA-FC, and TPE-FC CMPs. * is the side band of solid-state nuclear magnetic resonance spectroscopy (NMR).

1,1′-Diacetylferrocene (0.437 g, 1.62 mmol) and PDAT (0.24 g, 0.81 mmol) were mixed in a glassy flask and boiled at 180 °C in dimethyl sulfoxide (DMSO) (50 mL) under N_2_ for about three days. After that, the reaction was switched off and cooled to produce the black precipitate that was gained through filtration and consequently treated with Soxhlet extraction by using THF, chloroform, methanol, and acetone, respectively. In the last step, the obtained black powder was dried in the furnace at 120 °C under vacuum for about 24 h (Scheme 1a). FTIR (KBr, cm^−1^, Figure 1a): 2907 (C–H aliphatic), 1649 (C=N), 1202 (C–N), 1379 and 1042 (C=C stretching of ferrocene unit).

### 2.7. Preparation of TPA-FC CMP

1,1′-Diacetylferrocene (0.437 g, 1.62 mmol) and TPA-NH_2_ (0.24 g, 0.82 mmol) were mixed in a glass flask and boiled at 180 °C in dimethyl sulfoxide (DMSO) (50 mL) under N_2_ for about three days. After that, the reaction was switched off and cooled to produce the black precipitate that was gained through filtration and consequently treated with Soxhlet extraction by using THF, chloroform, methanol, and acetone, respectively. In the last step, the obtained black powder was dried in the furnace at 120 °C under vacuum for about 24 h (Scheme 1b). FTIR (KBr, cm^−1^, Figure 1a): 2907 (C–H aliphatic), 1649 (C=N), 1202 (C–N), 1379 and 1042 (C=C stretching of ferrocene unit).

### 2.8. Preparation of TPE-FC CMP

1,1′-Diacetylferrocene (0.437 g, 1.62 mmol) and TPE-NH_2_ (0.24, 0.61 mmol) were mixed in a glass flask and boiled at 180 °C in dimethyl sulfoxide (DMSO) (50 mL) under N_2_ for about three days. After that, the reaction was switched off and cooled to produce the black precipitate that was gained through filtration and consequently treated with Soxhlet extraction by using THF, chloroform, methanol, and acetone, respectively. In the last step, the obtained black powder was dried in the furnace at 120 °C under vacuum for about 24 h (Scheme 1c). FTIR (KBr, cm^−1^, Figure 1a): 2907 (C–H aliphatic), 1649 (C=N), 1202 (C–N), 1379 and 1042 (C=C stretching of ferrocene unit).

## 3. Results

### 3.1. Characterization and Properties of Ferrocene-Conjugated Microporous Polymers 

A total of three conjugated microporous polymers containing a ferrocene unit were achieved through a Schiff base reaction of 1,1′-diacetyl ferrocene as building unit with PDAT, TPA-NH_2_, and TPE-NH_2_ in DMSO at 180 °C to result in PDAT-FC, TPA-FC, and TPE-FC CMPs, respectively (Scheme 1a–c). First, the 1,1′-diacetylferrocene compound was synthesized through the reaction of ferrocene with acetyl chloride and aluminum chloride in DCM as a solvent for 8 h to produce a deep-red solid (Scheme S1). The FTIR profile (Figure S1) of 1,1′-diacetylferrocene showed peaks at 1391 and 1011 due to the presence of cyclopentadiene rings. The ^1^H-NMR spectrum (Figure S2a) of 1,1′-diacetylferrocene showed the proton’s signal at 4.79, 4.59, and 2.28 ppm, which were attributed to cyclopentadiene rings and methyl group for the acetyl unit, respectively. The presence of the C=O group in the 1,1′-diacetylferrocene monomer was confirmed by ^13^C-NMR analysis (Figure S2b). Second, the PDAT monomer was prepared through the refluxing of 1,4-dicyanobenzene with 2-cyanoguanidine and potassium hydroxide in 80 mL of DMF at 150 °C for 20 h under nitrogen to a white powder (Scheme S2). The peaks at 3311 and 3135 cm^−1^ in the FTIR pattern (Figure S3) of the PDAT monomer are due to the presence of the NH_2_ group. The signal at 8.33 ppm and 6.83 ppm were for aromatic protons and NH_2_ unit in the PDAT structure (Figure S4a). Furthermore, the ^13^C-NMR analysis (Figure S4b) of the PDAT revealed the presence of the C=O unit and triazine and aromatic ring at 170.65, 168.46, 139.96, and 127.91 ppm, respectively. Third, the reduction of TPA-NO_2_ in the presence of Pd/C and hydrazine monohydrate in dioxane/ethanol produced TPA-NH_2_ as gray crystals. Finally, the TPE-NH_2_ monomer was prepared through the reaction of 4,4′-diaminobenzophenone with Sn in the presence of HCl solution (12 M) to produce a green solid (Scheme S3). The FTIR, ^1^H, and ^13^C NMR measurements confirmed the successful synthesis of TPE-NH_2_ (Figures S5 and S6). The acquired FC CMPs revealed good chemical stability and insolubility in popular organic solvents (e.g., acetone, methanol, ethanol, DMF, and dichloromethane), indicative of their high degree of cross-linking. The structures of the acquired FC CMPs (PDAT-FC, TPA-FC, and TPE-FC CMPs) were approved from FT-IR and solid-state ^13^C NMR spectra measurements. All the FT-IR profiles of FC CMPs revealed absorption peaks were 1037–1042 cm^–1^, which was attributed to C=C stretching for the ferrocene unit. Significantly, the absorptions that were located around 1633 and 1200 cm^−1^ were assigned to (C=N) and (C–N) groups. Furthermore, characteristic vibration bands for the aliphatic C–H group were observed at 2925 cm^−1^ from the acetyl group of the ferrocene unit (Figure 1a). The ^13^C NMR profiles (Figure 1b) of PDAT-FC, TPA-FC, and TPE-FC CMPs produced signals at 37 ppm for the terminal acetyl group and signals from 77 to 89 were assigned to ferrocene moieties [54]. Intense signals were observed between 127 and 136 ppm, ascribed to the carbon atoms in aromatic nuclei. Additionally, a signal appeared at 146 ppm and could be linked to the C=N group. Moreover, PDAT-FC CMP featured an extra signal at 164 ppm for the triazine ring (Figure 1b). The thermal properties of FC CMPs were checked by using thermogravimetric analysis (TGA) measurements under a nitrogen atmosphere (Figure 1c, Table 1). TGA profiles of PDAT-FC, TPA-FC, and TPE-FC CMPs confirmed that they are chemically stable with decomposition temperatures of 99, 117, and 96 °C, respectively, at 5 wt%, as well as decomposition temperatures of 157, 197, and 174 °C, respectively, at 10 wt%. Moreover, the residual weights were 34, 56, and 51 wt% at 800 °C for PDAT-FC, TPA-FC, and TPE-FC CMPs, respectively. Powder X-ray diffraction measurements (Figure 1d and Figures S7–S9) reflected a broad diffraction peak for the PDAT-FC sample, demonstrating its amorphous feature, while the other two samples (TPA-FC and TPE-FC CMPs) presented some semi-crystalline peaks in addition to their broad diffraction peaks, as shown in their XRD profiles [54].

**Figure 2:**
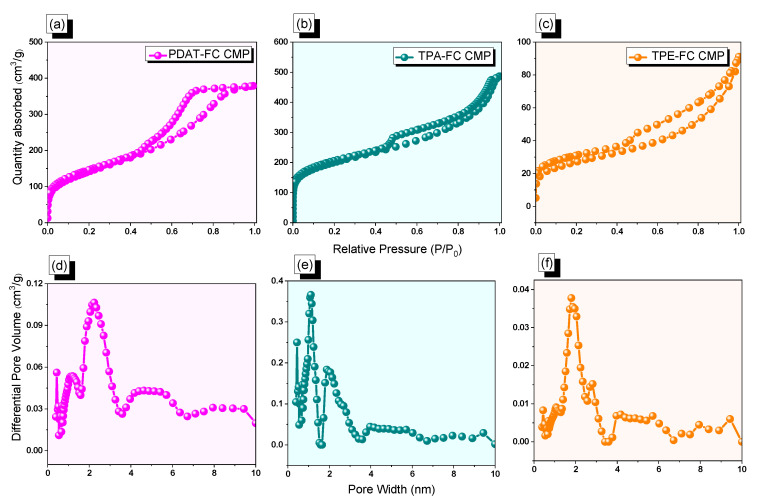
(**a**–**c**) N_2_ adsorption/desorption and (**d–f**) pore size profiles of PDAT-FC (**a**,**d**), TPA-FC (**b**,**e**), and TPE-FC CMPs (**c**,**f**).

The porous properties of FC CMPs (PDAT-FC, TPA-FC, and TPE-FC CMPs) were probed via nitrogen sorption measurements (adsorption/desorption isotherms) at 77 K. As illustrated in Figure 2a–c, these three FC CMP samples featured similar adsorption curves (Type IV) of nitrogen adsorption isotherms, showing the existence of both micropores and mesopores (hysteresis loops) within the polymeric networks, with BET surface area values of 502, 701, and 100 m^2^ g^−1^, respectively, for PDAT-FC, TPA-FC, and TPE-FC CMPs. Moreover, Figure 2d–f revealed the pore size distribution curves of the three samples, as calculated from nonlocal DFT, which is coordinated with adsorption/desorption profiles. Notably, the PDAT-FC CMP sample revealed a pore size diameter of 1.11–4.80 nm, while TPA-FC CMP reflected a diameter of 1.16–3.90 nm, and TPE-FC CMP was 1.83–4.10 nm (Table 1). 

**Figure 3:**
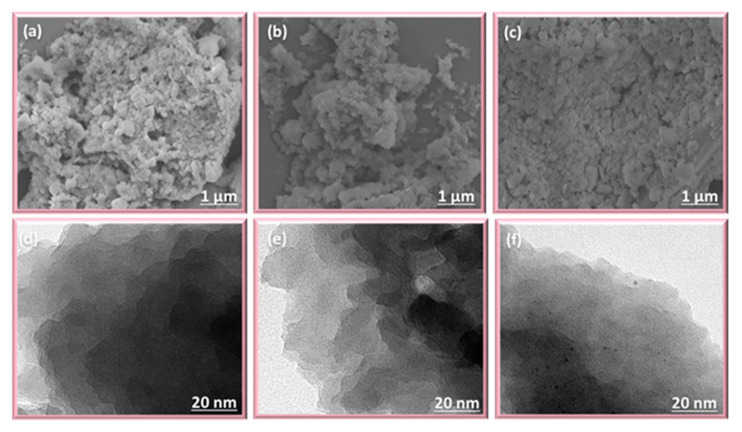
(**a**–**c**) SEM N_2_ and (**d**–**f**) TEM images of PDAT-FC, TPA-FC, and TPE-FC CMPs, respectively.

The morphology of FC CMPs is investigated from SEM and TEM microscopic techniques (Figure 3). These three materials featured an aggregation of irregular nanoparticles, as demonstrated in SEM images (Figure 3a–c), as well as they featured disordered structures as confirmed by TEM images (Figure 3d–f) [62].

### 3.2. Electrochemical Analysis of Ferrocene-Conjugated Microporous Polymers

**Figure 4:**
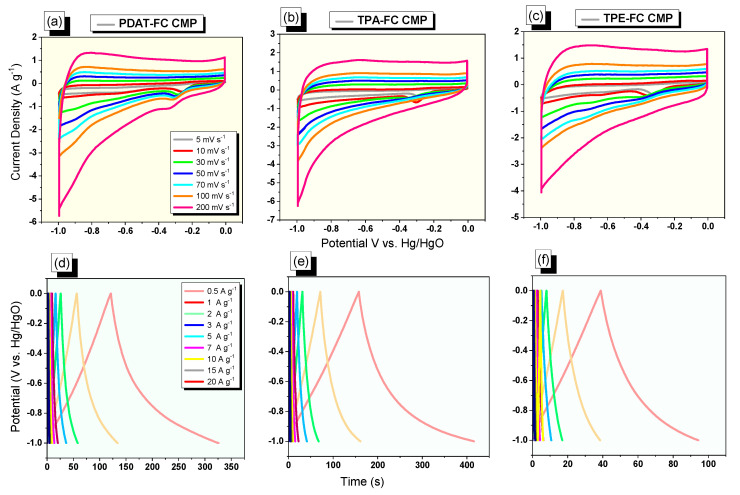
(**a**–**c**) CV and (**d**–**f**) GCD of PDAT-FC (**a**,**d**), TPA-FC (**b**,**e**), and TPE-FC CMPs (**c**,**f**).

The electrochemical capability of ferrocene-conjugated microporous polymers (FC CMPs) was examined via a three-electrode supercapacitor cell in an aqueous KOH solution (1 M) as the electrolyte. First, cyclic voltammetry (CV) experiments of the three CMPs (PDAT-FC, TPA-FC, and TPE-FC CMPs) were done at sweep rates between 5 and 200 mV s^−1^, by applying a potential window from −1 to 0 V (Figure 4a–c). CVs of these three FC CMPs displayed almost rectangular shapes, with a significant faradic peak appearing around −0.33 V, revealing pseudocapacitance behavior that arises from redox reactions that occur in all CMPs due to the coexistence of a nitrogen heteroatom and ferrocene units. Notably, there is an excellent linear relationship that is observed between current densities and sweep rates from 5 to 200 mV s^−1^, suggesting that all FC CMPs have typical rate capabilities and stabilities. Also, galvanostatic charge/discharge (GCD) tests were done for PDAT-FC, TPA-FC, and TPE-FC CMPs, at the same voltage range (−1 to 0 V) using a current density range starting from 0.5 to 20 A g^−1^, to expect the capacitive performance. These three CMPs appeared as symmetrical triangle shapes in their GCD curves (Figure 4d–f), and we can notice the rapid charge and discharge times, suggesting higher specific capacitance values for the three FC CMPs. However, the TPA-FC CMP material featured a more extended discharge time compared with the other two CMPs, showing its good capacitive performance at all current densities.

**Figure 5:**
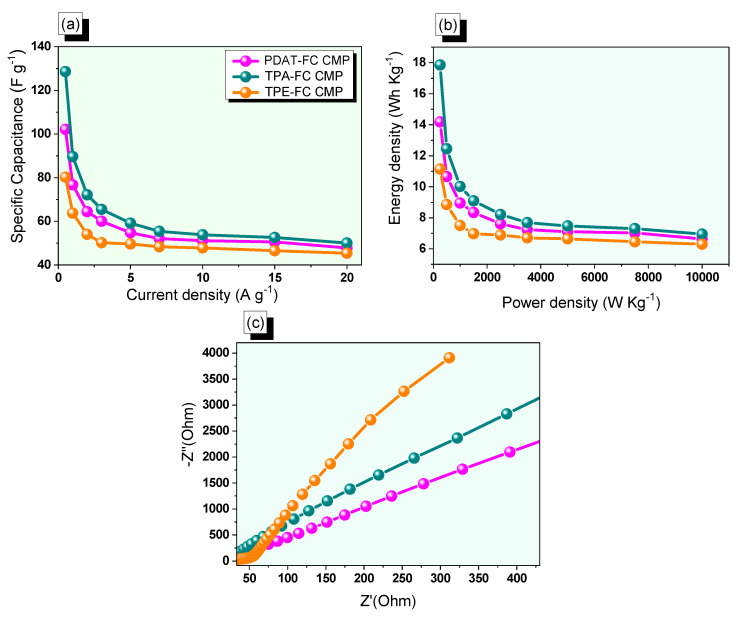
(**a**) Specific capacitance, (**b**) Ragone, and (**c**) Nyquist profiles of PDAT-FC, TPA-FC, and TPE-FC CMPs.

Based on their discharge time, the specific capacitance values of PDAT-FC, TPA-FC, and TPE-FC CMPs were calculated at all current density ranges (Figure 5a). Notably, the capacitance values for PDAT-FC CMP were recorded to be 102, 77, 64, 60, 55, 52, 51, 50, and 47 F g^−1^, respectively, from 0.5 to 20 A g^−1^. The corresponding values for TPA-FC CMP were 129, 90, 72, 65, 59, 55, 53, 52, and 50 F g^−1^, respectively, at the same current density range. While TPE-FC CMP displayed capacitances of 80, 64, 54, 50, 49, 48, 47, 46, and 45 F g^−1^, respectively. Notably, the highest capacitance of TPA-FC CMP (129 F g^−1^) compared with the other samples is related to the presence of redox-active triphenylamine and ferrocene units in its backbone [59,63,64,65], in addition to a high surface area and good porosity that facilitates the redox process and provides rapid kinetics. We summarized the capacity values of our FC CMP samples and compared them with other reported data of three electrode supercapacitor materials (Table S1) [59,66,67,68,69,70,71,72,73]. Moreover, Ragone plots that combine the energy and power density together are considered beneficial indicators for determining the efficiency of supercapacitors. According to Figure 5b, the PDAT-FC sample offers an energy density of 14 Wh kg^−1^, while TPA-FC was 18 Wh kg^−1^ and TPE-FC showed 11 Wh kg^−1^, respectively, working at a power density value of 250 W kg^−1^. Electrochemical impedance spectroscopy (EIS) for the FC CMPs was measured to study the ion transport properties and the effect of the presence of ferrocene moieties on their specific capacitance. Figure 5c and Figure S10 display Nyquist plots and their corresponding identical fitted circuits to study various parameters such as charge transfer resistances and series of electrodes. The fitted circuit (Figure S10a) displayed the parallel series of resistance (R_s_), charge transfer resistance (R_ct_), and constant phase elements (CPE-EDL and CPE-P) which are each symbolized by CPE1 and CPE2, in addition to the Warburg element (w1), respectively. As presented in Figure S10b, PDAT-FC, TPA-FC, and TPE-FC CMPs had charge transfer resistances of 10.8, 14.26, and 74.59 Ω, respectively. From the Nyquist plots (Figure 5c), TPA-FC CMP featured the lowest impedance (i.e., resistance) compared with the other samples, showing its rapid charge transfer through the electrode and electrolyte interface, resulting from the outstanding redox activity of TPA and ferrocene units [59,63,64,65]. Notably, the curve of the TPA-FC CMP appeared nearly perpendicular in the low-frequency region to the vertical axis, revealing the rapid movement of ions across the electrode surface.

**Figure 6:**
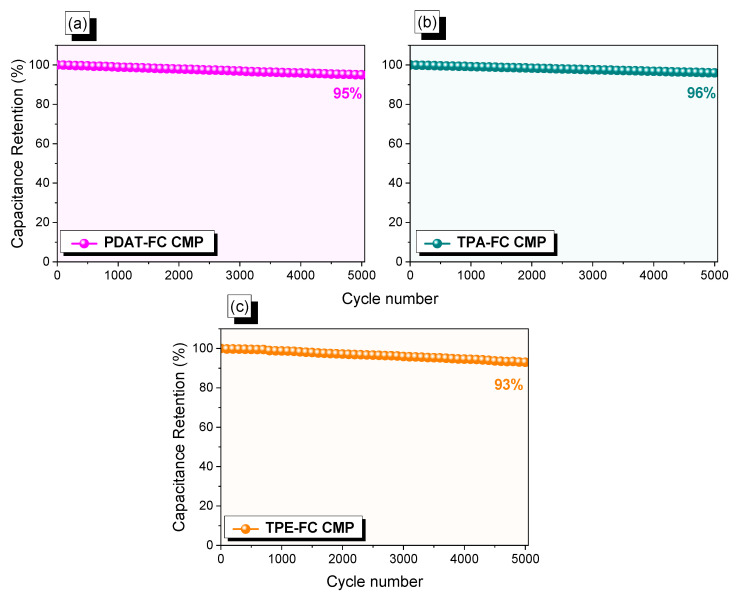
Stability profiles at 5000 cycles for PDAT-FC (**a**), TPA-FC (**b**), and TPE-FC CMPs (**c**).

Further, from Figure 6a–c, the FC CMPs electrodes featured remarkable cycle stability over 5000 cycles at higher current density (10 A g^−1^), resulting in capacitance retention values of 95%, 96%, and 93% for PDAT-FC, TPA-FC, and TPE-FC CMPs, respectively. Additionally, CR2032 coin cells were assembled to investigate the electrochemical efficiency of PDAT-FC, TPA-FC, and TPE-FC CMPs SSCs, using a potential range of −0.7 to +0.3 V. We performed the CV profiles of the PDAT-FC, TPA-FC, and TPE-FC CMPs at sweep rates between 5 and 200 mV s^−1^ (Figure S11a–c). The CV profiles of all FC CMPs offered nearly rectangular shapes. Figure S11d–f also demonstrates the GCD curves of the PDAT-FC, TPA-FC, and TPE-FC CMPs SSCs electrodes, respectively, done at numerous current densities from 0.5 to 10 A g^−1^. The curves appeared almost triangular shaped, that reflect the influence of electric double-layer capacitance and pseudocapacitance. At a current density of 0.5 A g^−1^, the specific capacitances of PDAT-FC, TPA-FC, and TPE-FC CMPs electrodes were calculated via their GCD curves, to be 52, 65, and 34 F g^−1^, respectively (Figure S12a). Notably, the capacitance of the TPA-FC CMP electrode was the highest compared with PDAT-FC and TPE-FC CMPs, because of the presence of redox-active triphenylamine and ferrocene units in its backbone, in addition to its high surface area and good porosity that facilitates the redox process and provides rapid kinetics, leading to the best performance of the TPA-FC CMP material. Moreover, Ragone plots of all FC CMPs SSCs were recorded to evaluate the energy density of the materials, which determined the efficiency of SSCs. According to Figure S12b, the PDAT-FC electrode displayed an energy density of 7 Wh kg^−1^, while TPA-FC offered 9 Wh kg^−1^, and TPE-FC showed 5 Wh kg^−1^, respectively, at a power density value of 250 W kg^−1^.

## 4. Conclusions

In summary, facile conjugated microporous polymers (FC CMPs) were presented by integrating the ferrocene unit with different aryl amine monomers through a Schiff base reaction to produce PDAT-FC, TPA-FC, and TPE-FC CMPs, respectively, as supercapacitor electrodes. The TPA-FC CMP sample achieved the highest surface area of about 701 m^2^ g^−1^, high char yield of 56 wt% at 800 °C as well as a good capacitive performance with a specific capacitance of 129 F g^−1^ and capacitance retention value of 96% over 5000 cycles, was accomplished compared with the other two materials. This feature of TPA-FC CMP is attributed to redox-active triphenylamine and ferrocene units in its backbone that facilitate the redox process and provide rapid kinetics. Our study suggests promising materials for constructing efficient electrodes that could be utilized in supercapacitor applications. Furthermore, these CMPs materials can be applied for gas capture and dye adsorption.

## Figures and Tables

**Table 1:** TGA and pore parameters data for PDAT-FC, TPA-FC, and TPE-FC CMPs.

Sample	*T*_d5_ (°C)	*T*_d10_ (°C)	Char Yield (wt%)	Surface Area (m^2^ g^−1^)	Pore Size (nm)
PDAT-FC CMP	99	157	34	502	1.11, 2.24, 4.80
TPA-FC CMP	117	197	56	701	1.16, 1.07, 3.99
TPE-FC CMP	96	174	51	100	1.83, 2.86, 4.10

## Data Availability

The data that were presented in this study are available on request from the corresponding author.

## Supplementary Materials

The following supporting information can be downloaded at: https://www.mdpi.com/article/10.3390/polym15051095/s1, Scheme S1: Preparation method of 1,1′-diacetylferrocene; Scheme S2: Preparation method of 1,4-bis(4,6-diamino-s-triazin-2-yl)benzene (PDAT); Scheme S3: Preparation method of TPE-NH_2_; Figure S1: FT-IR profile of 1,1′-diacetylferrocene; Figure S2: (a) ^1^H-NMR profile and (b) ^13^C-NMR profile of 1,1′-diacetylferrocene; Figure S3: FT-IR profile of 1,4-bis(4,6-diamino-s-triazin-2-yl)benzene (PDAT); Figure S4: (a) ^1^H-NMR profile and (b) ^13^C-NMR profile of 1,4-bis(4,6-diamino-s-triazin-2-yl)benzene (PDAT); Figure S5: FT-IR profile of TPE-NH_2_; Figure S6: (a) ^1^H-NMR profile and (b) ^13^C-NMR profile of TPE-NH_2_; Figure S7: XRD profile of PDAT-FC CMP; Figure S8: XRD profile of TPA-FC CMP; Figure S9: XRD profile of TPE-FC CMP; Figure S10: (a) Fitted circuits and (b) Table of the various parameters of the Nyquist plots; Figure S11: (a–c) CV and (d–f) curves of SSC coin cells incorporating the PDAT-FC (a,d), TPA-FC (b,e), and TPE-FC CMPs (c,f); Figure S12: (a) Specific capacitances and (b) Ragone profiles of SSC coin cells incorporating the PDAT-FC, TPA-FC, and TPE-FC CMPs; Table S1: Comparison between the capacity values of FC-CMPs samples with different reported data of three electrode supercapacitor materials [59,66,67,68,69,70,71,72,73].
