# Supplementary material for: Conjugated Microporous Polymers Based on Ferrocene Units as Highly Efficient Electrodes for Energy Storage
Source: Polymers (Basel). 2023 Feb 22;15(5):1095. doi: 10.3390/polym15051095 (PMC10007016; doi:10.3390/polym15051095)
Supplement: Supplementary file 1 [file polymers-15-01095-s001.zip › polymers-2186635-supplementary.pdf]

Supplementary Materials

Characterization

FTIR spectra were collected on a Bruker Tensor 27 FTIR spectrophotometer with a resolution of 4 cm^{-1} by using KBr disk method. ^{13}C nuclear magnetic resonance (NMR) spectra were examined by using an INOVA 500 instrument with DMSO as the solvent and TMS as the external standard. Chemical shifts are reported in parts per million (ppm). The thermal stabilities of the samples were performed by using a TG Q-50 thermogravimetric analyzer under a N_2 atmosphere; the cured sample (ca. 5 mg) was put in a Pt cell with heating rate of 20 °C min^{-1} from 100 to 800 °C under a N_2 flow rate of 60 mL min^{-1}. Wide-angle X-ray diffraction (WAXD) patterns were measured by the wiggler beamline BL17A1 of the National Synchrotron Radiation Research Center (NSRRC), Taiwan. A triangular bent Si (111) single crystal was used to get a monochromated beam having a wavelength (λ) of 1.33 Å. The morphologies of the polymer samples were examined by Field emission scanning electron microscopy (FE-SEM; JEOL JSM7610F) and also by transmission electron microscope (TEM) using a JEOL-2100 instrument at an accelerating voltage of 200 kV. BET surface area and porosimetry measurements of samples (ca. 40–100 mg) were measured using BEL Master™/BEL sim™ (v. 3.0.0). N_2 adsorption and desorption isotherms were generated through incremental exposure to ultrahigh-purity N_2 (up to ca. 1 atm) in a liquid N_2 (77 K) bath. Surface parameters were calculated using BET adsorption models in the instrument's software. The pore size of the prepared samples was determined by using nonlocal density functional theory (NLDFT).

Electrochemical Analysis

Working Electrode Cleaning: Prior to using, the glassy carbon electrode (GCE) was polished several times with 0.05-μm alumina powder, washed with EtOH after each polishing step, cleaned through sonication (5 min) in a water bath, washed with EtOH, and then dried in air.

Electrochemical Characterization: The electrochemical experiments were performed in a three-electrode cell using an Autolab potentiostat (PGSTAT204) and 1 M KOH as the aqueous electrolyte. The GCE was used as the working electrode (diameter: 5.61 mm; 0.2475 cm^2); a Pt wire was used as the counter electrode; Hg/HgO (RE-1B, BAS) was the reference electrode. All reported potentials refer to the Hg/HgO potential. A slurry was prepared by dispersing FC-CMPs (45 wt %), carbon black (45 wt %) and Nafion (10 wt %) in a mixture of (EtOH/ H_2O) (200 μL: 800 μL) and then sonicated for 1 h. A portion of this slurry (10 μL) was pipetted onto the tip of the electrode, which was then dried in air for 30 min prior to use. The electrochemical performance was studied through CV at various sweep rates (5–200 mV s^{-1}) and through the GCD method in the potential range from 0 to -1.00 V (vs. Hg/HgO) at various current densities (0.5–20 A g^{-1}) in 1 M KOH as the aqueous electrolyte solution.

The specific capacitance was calculated from the GCD data using the equation.

$$C_s = (I\Delta t)/(m\Delta V) \tag{S1}$$

Where C_s (F g^{-1}) is the specific capacitance of the supercapacitor, I (A) is the discharge current, ΔV (V) is the potential window, Δt (s) is the discharge time, and m (g) is the mass of the NPC on the electrode. The energy density (E, W h kg^{-1}) and power density (P, W kg^{-1}) were calculated using the equations.

$$E = 1000C(\Delta V)^2/(2 \times 3600) \tag{S2}$$

$$P = E/(t/3600) \tag{S3}$$

We evaluated the electrochemical functionality of a symmetric supercapacitor using a CR2032 coin cell, which consists of an anode and cathode, a bottom and top cover, a metal spring, a separator, and an electrolyte. Our compounds served as both the cathode and the anode in order to construct a symmetric supercapacitor. The slurry was created

by combining 2 mg of CMP, 2 mg of conductive carbon, 20 mL of nafion, 200 mL of ethanol, and 400 mL of water. It was then sonicated for an hour and cast onto carbon paper. We used a Selemion AMV membrane with an electrolyte of 1.0 M aqueous KOH.

The specific capacitance was calculated in assembled supercapacitor from the GCD data using the following equations:

$$C_s = 2(I\Delta t)/(m\Delta V) \quad \text{(S4)}$$

where C_s (F g^{-1}) is the specific capacitance of the supercapacitor, I (A) is the discharge current, ΔV (V) is the potential window, Δt (s) is the discharge time, and m (g) is the mass of the CMP in a single electrode.

The energy density (E, W h kg^{-1}) and power density (P, W kg^{-1}) were calculated using the equations:

$$E_{cell} = 1000\ C_s\ (\Delta V)^2/(4 \times 2 \times 3600) \quad \text{(S5)}$$

And

$$P_{cell} = E_{cell}/(t/3600) \quad \text{(S6)}$$

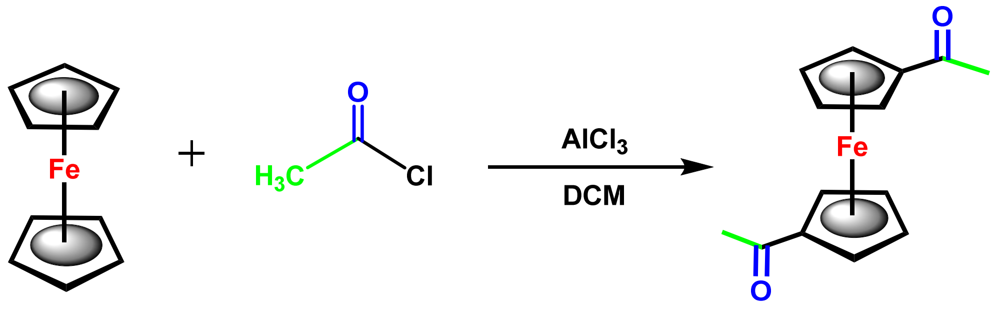

Scheme S1. Preparation method of 1,1'-diacetylferrocene.

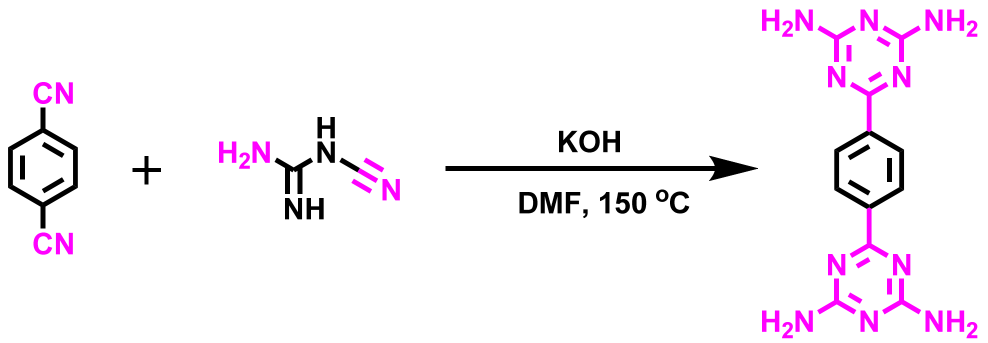

Scheme S2. Preparation method of 1,4-bis(4,6-diamino-s-triazin-2-yl)benzene (PDAT).

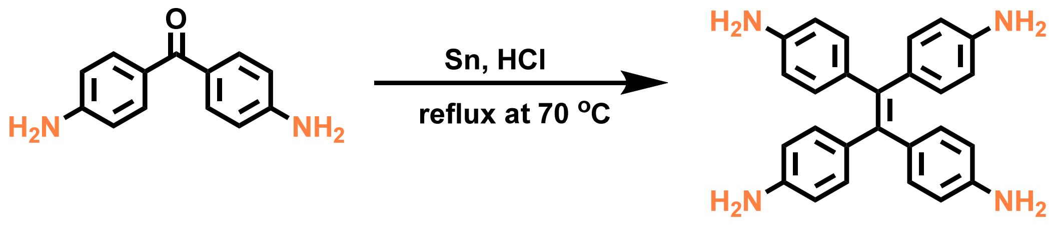

Scheme S3. Preparation method of TPE-NH_2.

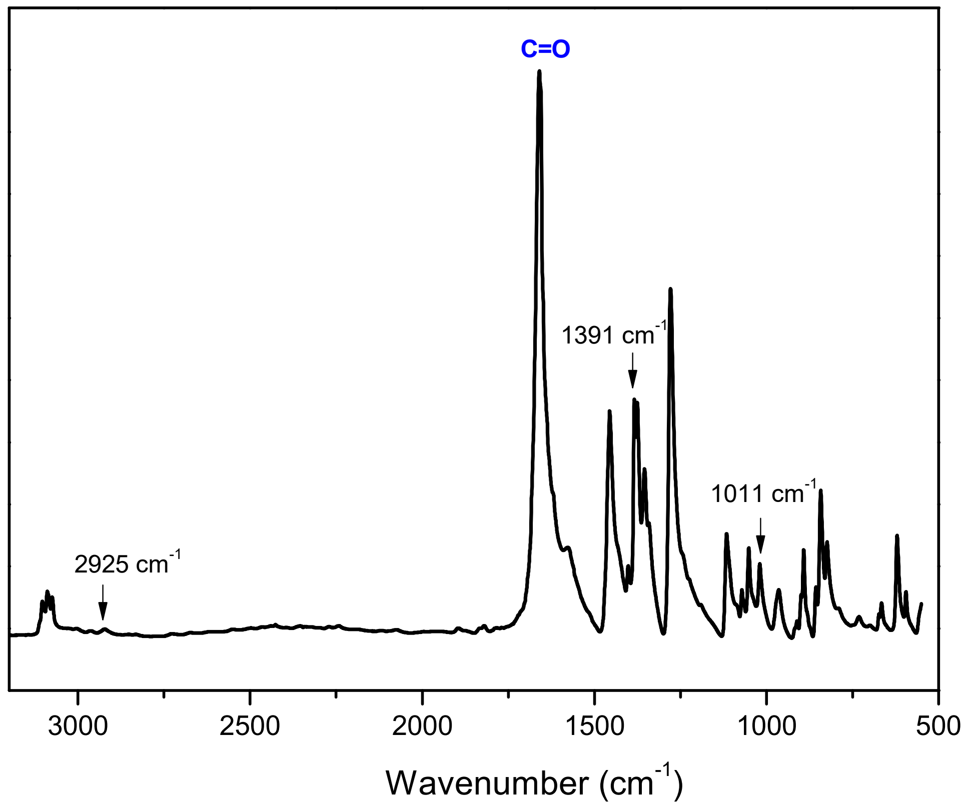

Figure S1. FT-IR profile of 1,1'-diacetylferrocene.

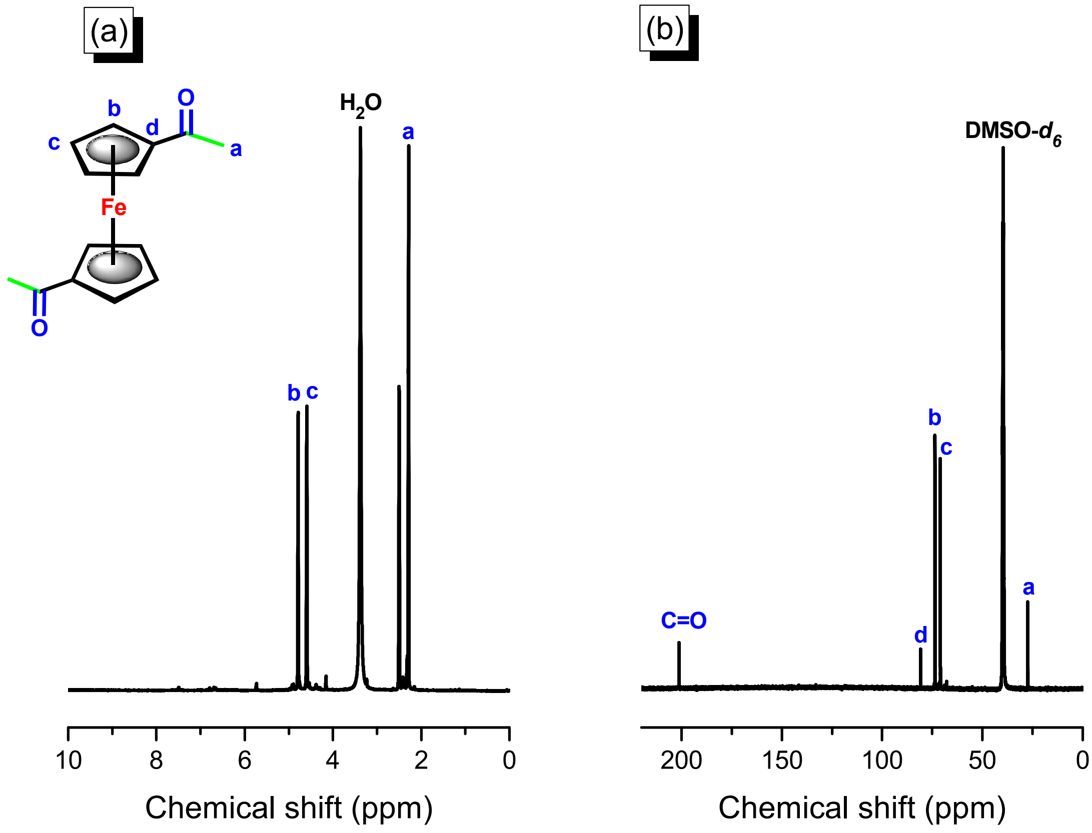

Figure S2. (**a**) ^{1}H-NMR profile and (**b**) ^{13}C-NMR profile of 1,1'-diacetylferrocene.

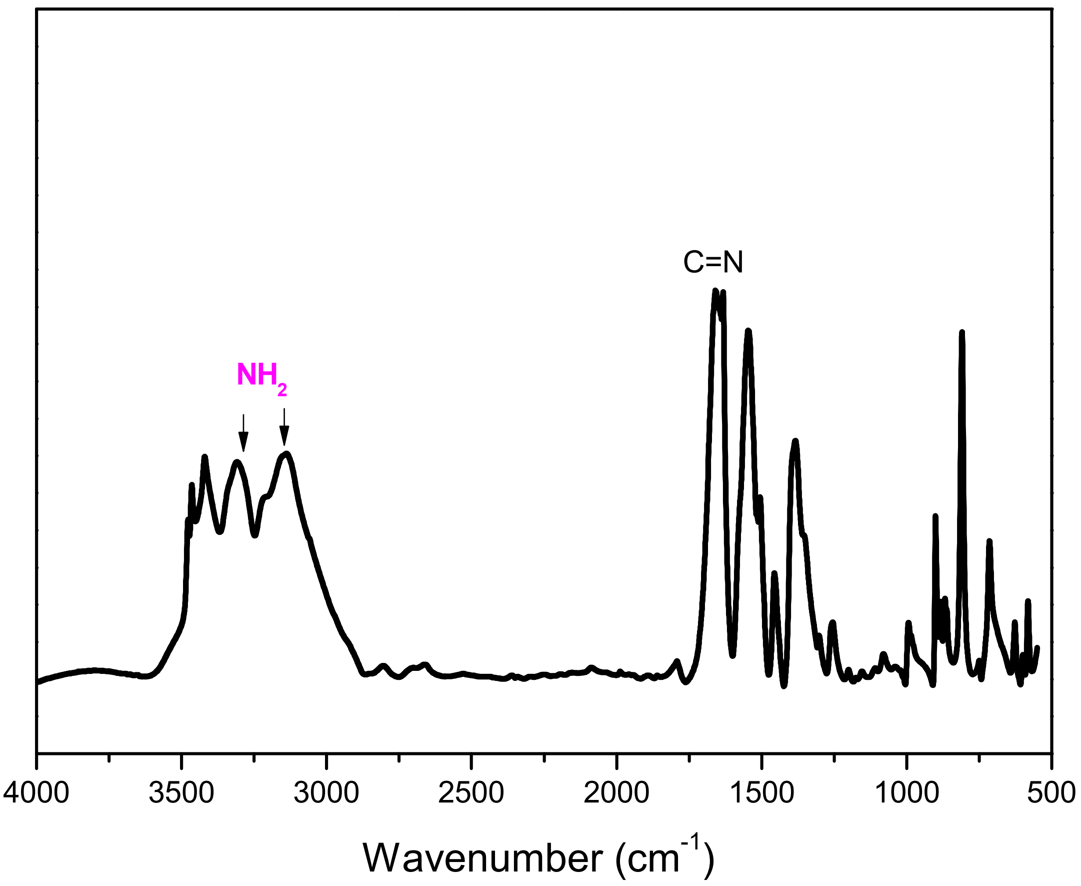

Figure S3. FT-IR profile of 1,4-bis(4,6-diamino-s-triazin-2-yl)benzene (PDAT).

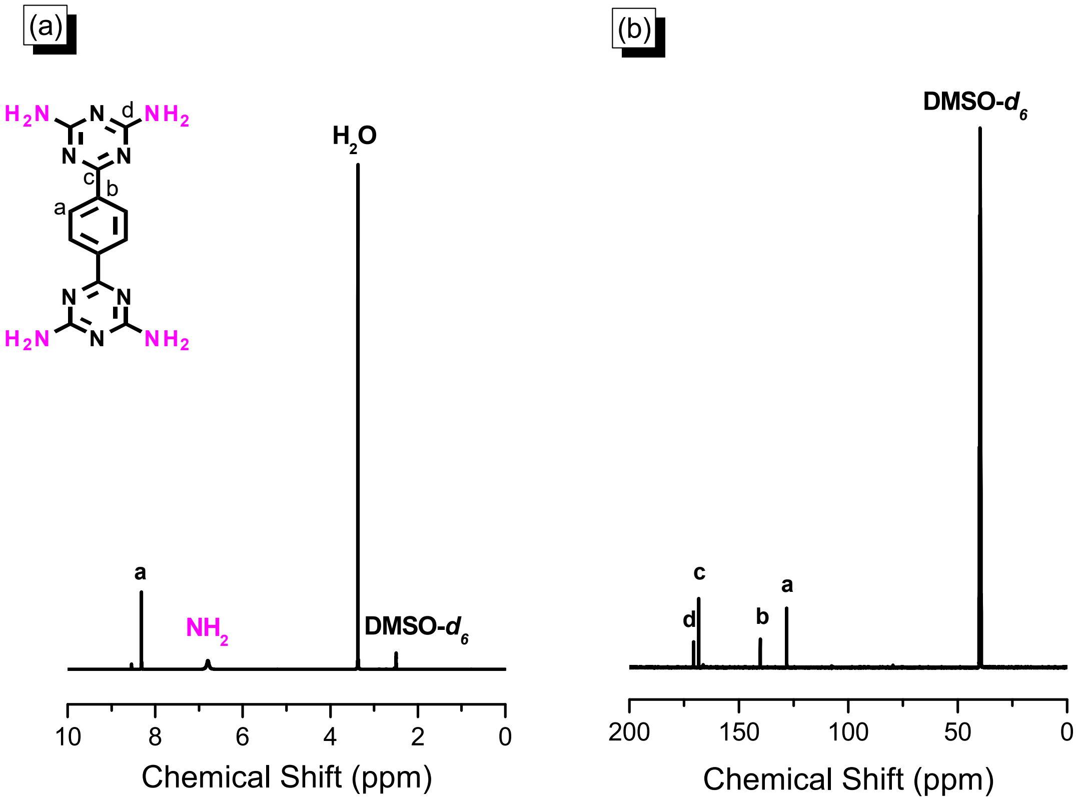

Figure S4. (**a**) ^{1}H-NMR profile and (**b**) ^{13}C-NMR profile of 1,4-bis(4,6-diamino-s-triazin-2-yl)benzene (PDAT).

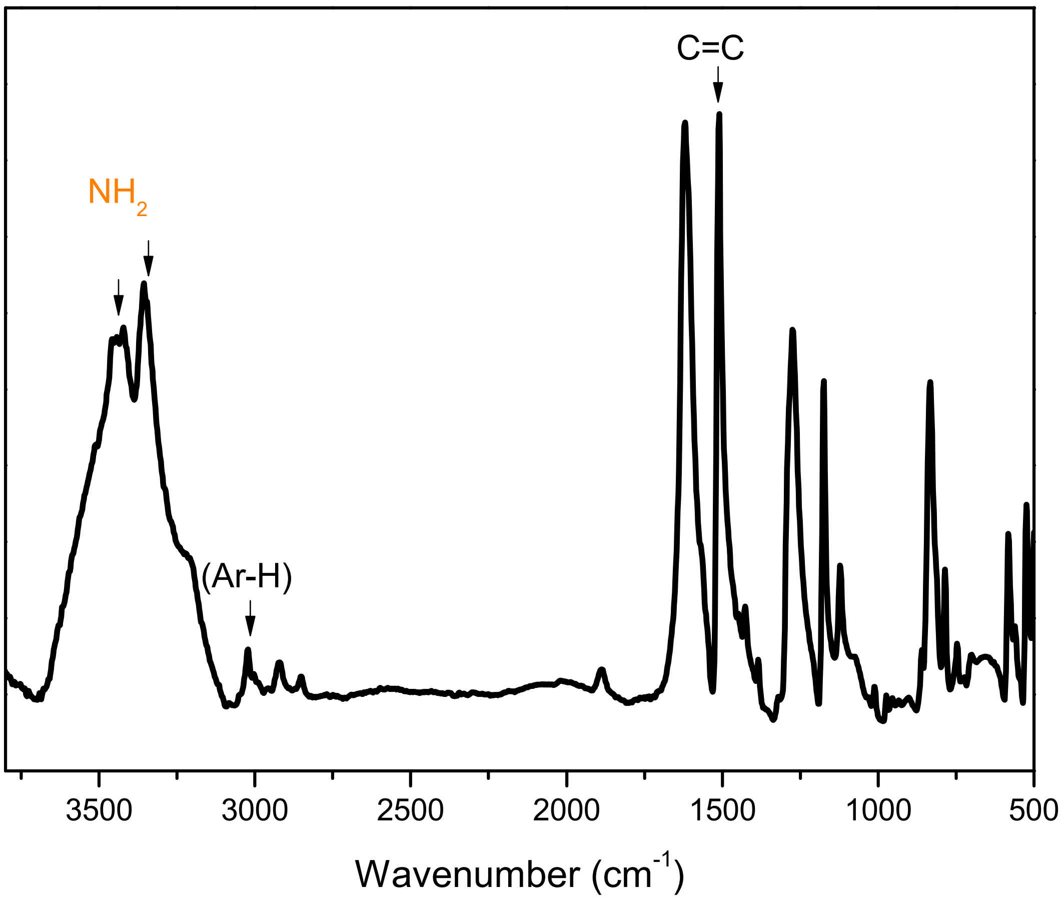

Figure S5. FT-IR profile of TPE-NH_2.

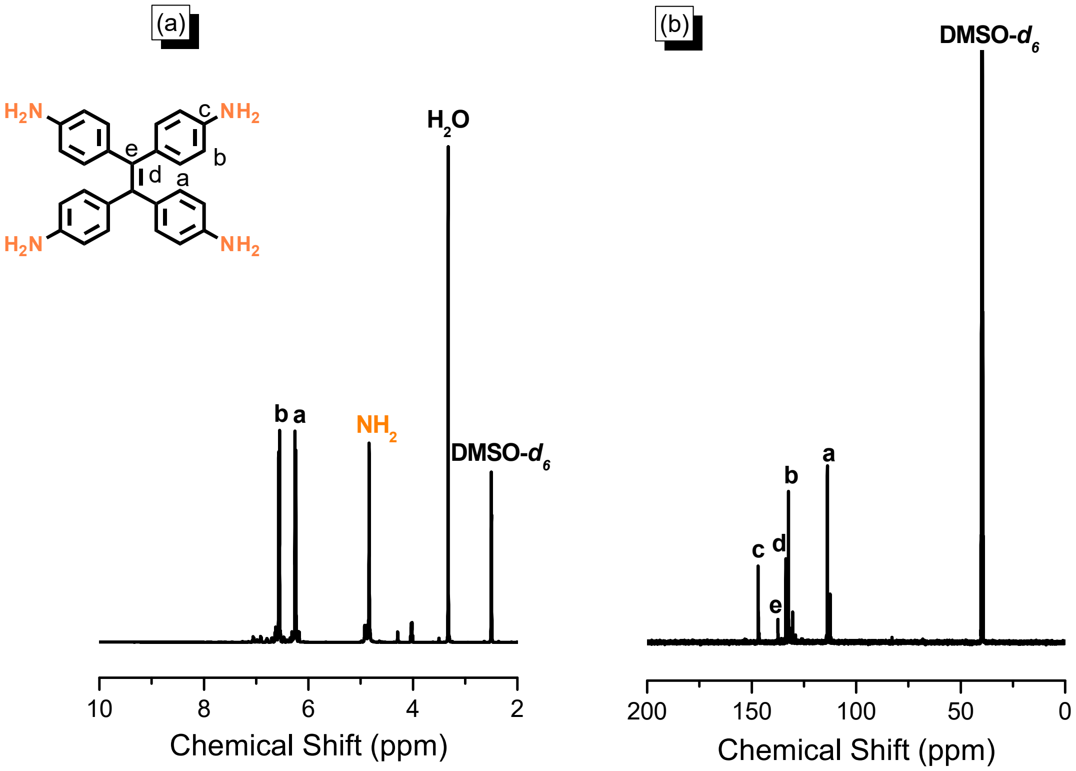

Figure S6. (**a**) ^{1}H-NMR profile and (**b**) ^{13}C-NMR profile of TPE-NH_2.

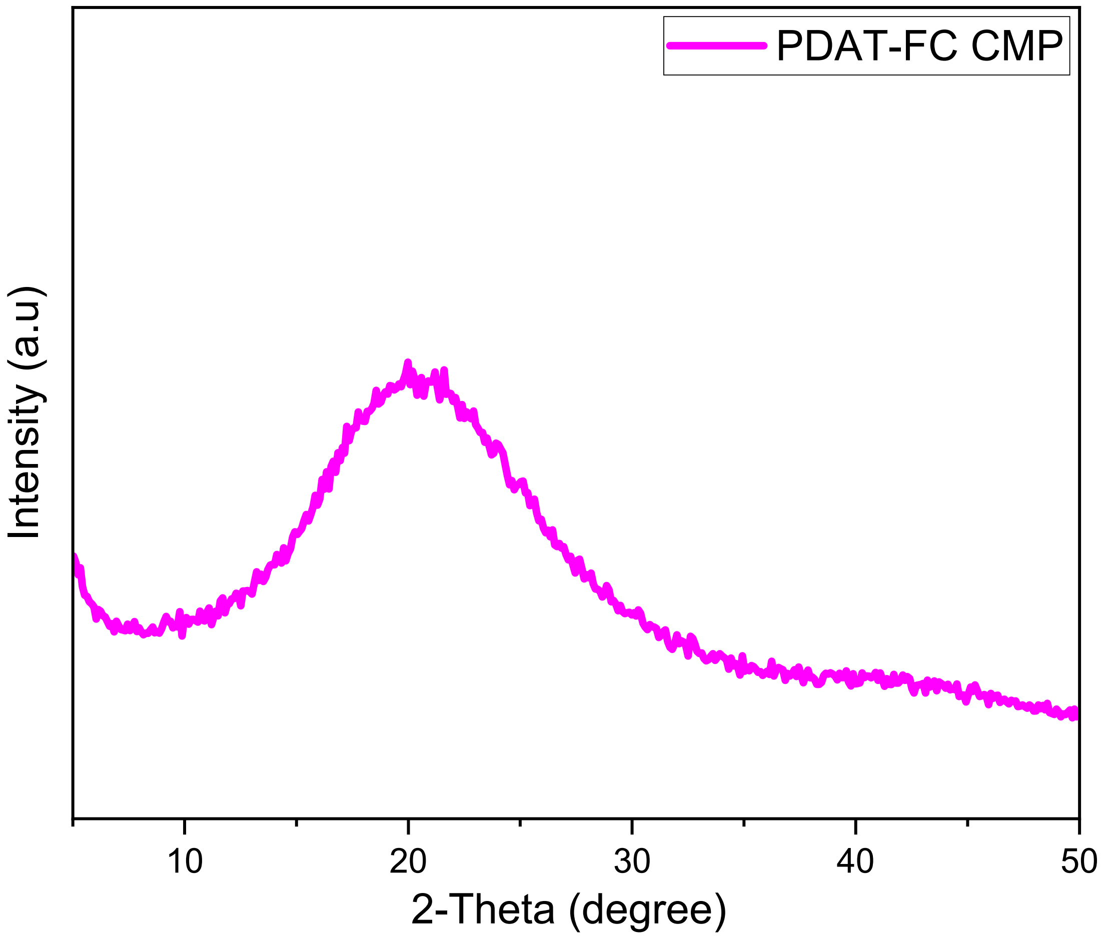

Figure S7. XRD profile of PDAT-FC CMP.

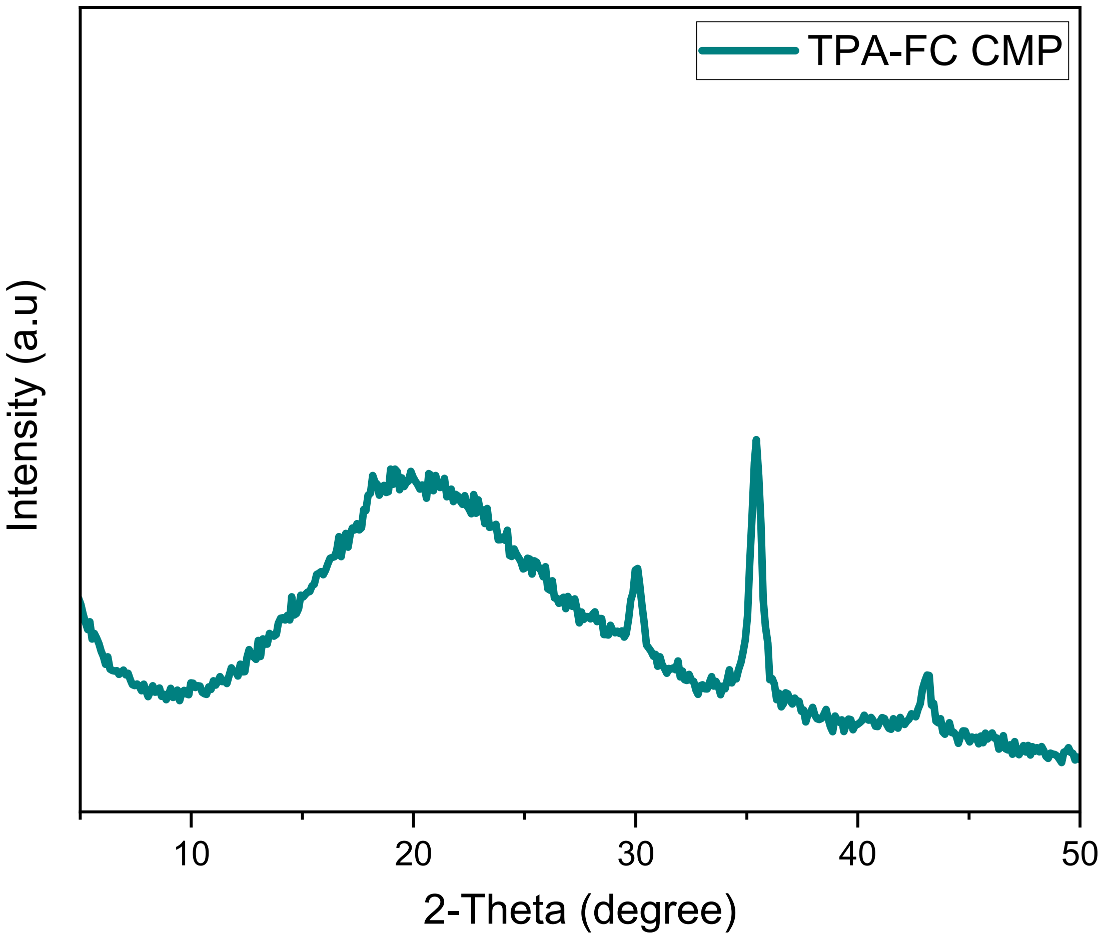

Figure S8. XRD profile of TPA-FC CMP.

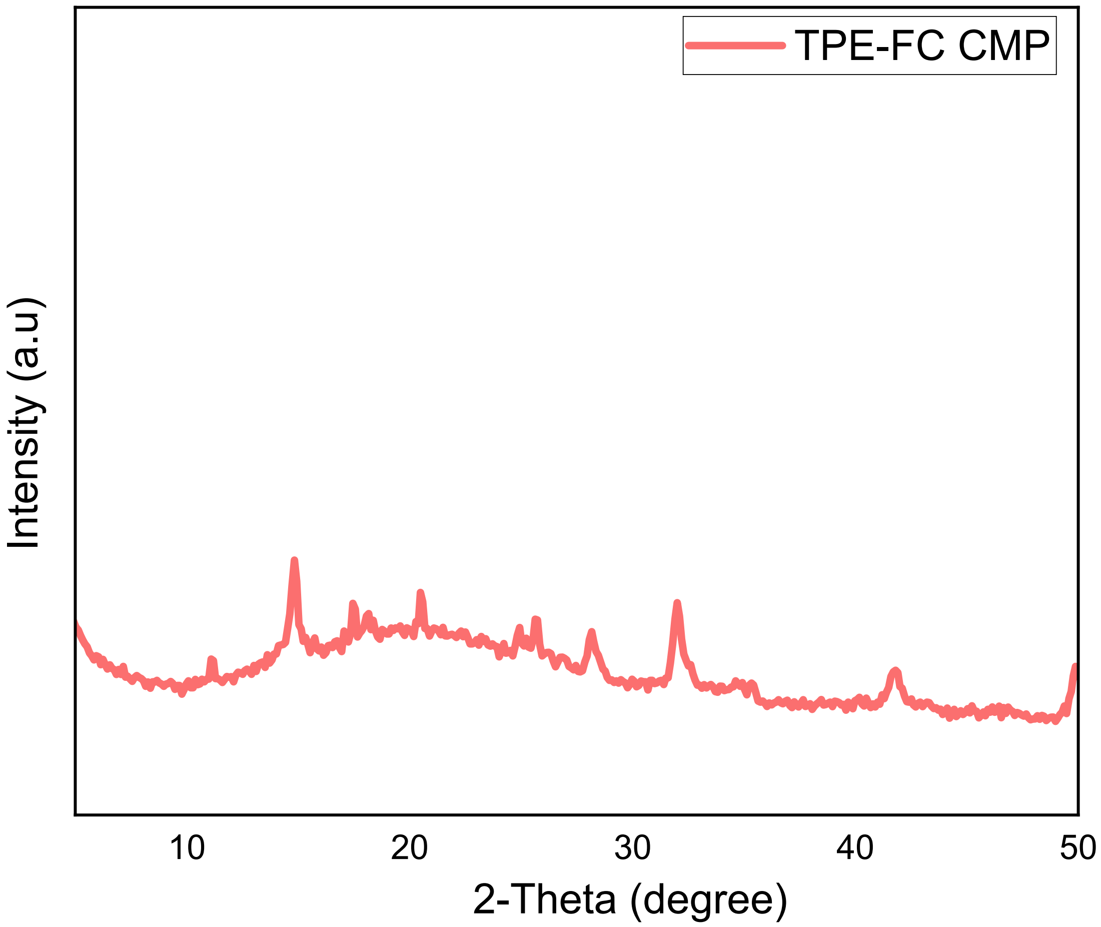

Figure S9. XRD profile of TPE-FC CMP.

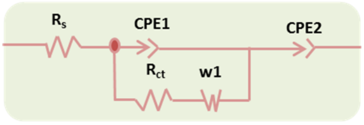

(**a**)

Sample	R_s (Ω)	R_{ct} (Ω)	CPE-EDL (S s^n)	CPE-P (S s^n)
PDAT-FC CMP	0.026661	14.26	4.1324×10^{-5}	0.47096
TPA-FC CMP	2.1806×10^{-7}	10.8	1.7428×10^{-4}	0.37874
TPE-FC CMP	0.0014366	74.59	2.2788×10^{-5}	0.91675

(**b**)

Figure S10. (**a**) Fitted circuits and (**b**) Table of the various parameters of the Nyquist plots.

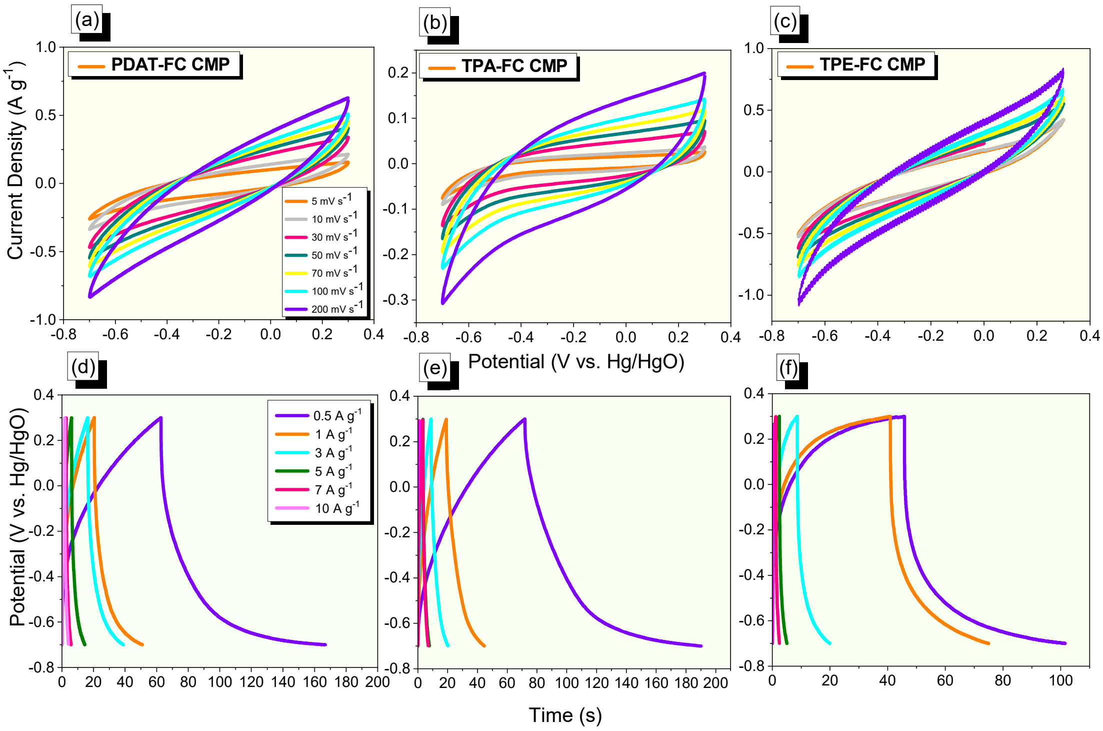

Figure S11. (**a**–**c**) CV and (**d**–**f**) curves of SSC coin cells incorporating the PDAT-FC (**a**,**d**), TPA-FC (**b**,**e**), and TPE-FC CMPs (**c**,**f**).

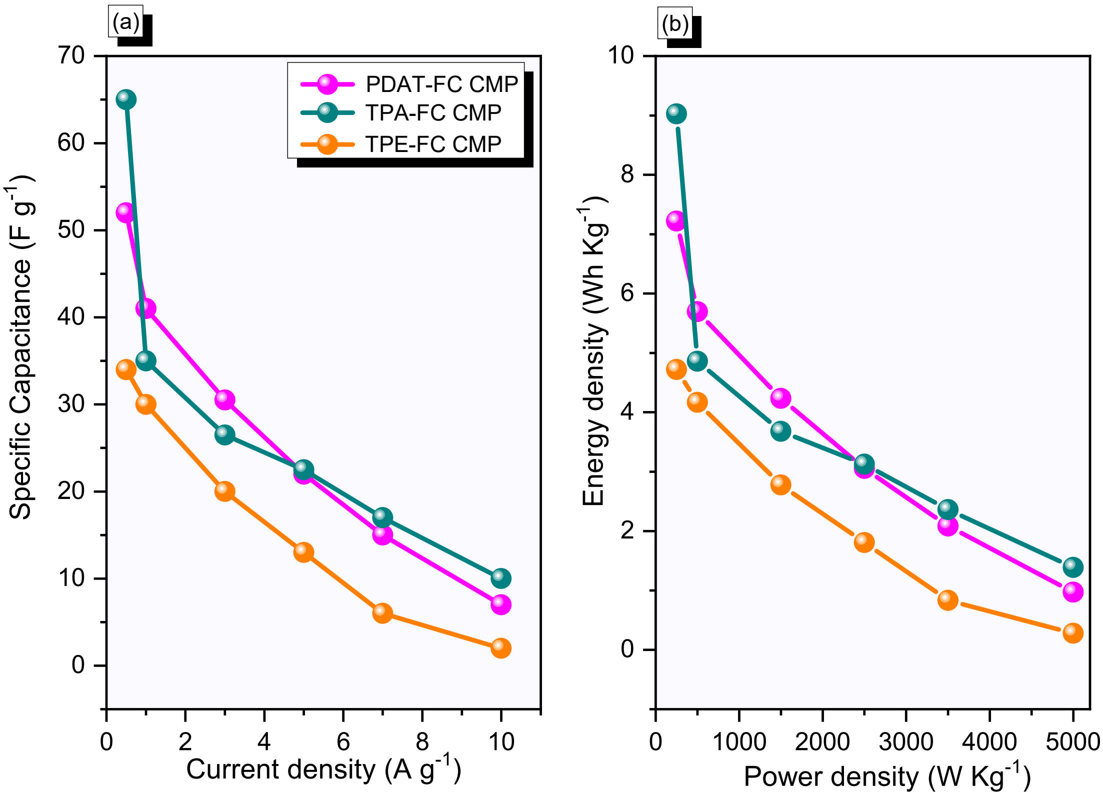

Figure S12. (**a**) Specific capacitances and (**b**) Ragone profiles of SSC coin cells incorporating the PDAT-FC, TPA-FC and TPE-FC CMPs.

Table S1. Comparison between the capacity values of FC-CMPs samples with different reported data of three electrode supercapacitor materials [60,67–74].

Electrode	Capacitance (Fg^{-1})	Ref
PDAT-FC CMP	102 F g^{-1} at 0.5 A g^{-1}	This work

TPA-FC CMP	129 F g^{-1} at 0.5 A g^{-1}	This work
TPE-FC CMP	80 F g^{-1} at 0.5 A g^{-1}	This work
CMPs	72 F g^{-1} at 0.5 A g^{-1}	[59]
CoPc-CMP	13.8 F g^{-1} at 1 A g^{-1}	[66]
β-Ketoenamine-Linked Covalent Organic Frameworks (COF)	48 F g^{-1} at 0.1 A g^{-1}	[67]
Conductive MOF	111 F g^{-1} at 0.05 A g^{-1}	[68]
POSS-F-POIP	36.2 F g^{-1} at 0.5 A g^{-1}	[69]
TBN-Py-CMP	31 F g^{-1} at 0.5 A g^{-1}	[70]
TBN-TPE-CMP	18.45 F g^{-1} at 0.5 A g^{-1}	[70]
TBN-Car-CMP	18.90 F g^{-1} at 0.5 A g^{-1}	[70]
Activated carbon from waste Compact Discs (CDs)	51 F g^{-1} at 10 mV s^{-1}	[71]
N-doped Porous carbons ropes	60 F g^{-1} at 1.0 A g^{-1}	[72]
Pyrolysis of pistachio nutshell	45 F g^{-1} at 0.5 A g^{-1}	[73]
